# Macular Hemorrhage Due to Age-Related Macular Degeneration or Retinal Arterial Macroaneurysm: Predictive Factors of Surgical Outcome

**DOI:** 10.3390/jcm10245787

**Published:** 2021-12-10

**Authors:** Mitta Pierre, Adam Mainguy, Irini Chatziralli, Kaivon Pakzad-Vaezi, Jorge Ruiz-Medrano, Bahram Bodaghi, Anat Loewenstein, Jayakrishna Ambati, Marc D. de Smet, Ramin Tadayoni, Sara Touhami

**Affiliations:** 1 Department of Ophthalmology, Hôpital Lariboisière, Assistance Publique-Hôpitaux de Paris, Université de Paris, 75010 Paris, France; mitta.pierre@live.fr (M.P.); ramin.tadayoni@aphp.fr (R.T.); 2 Department of Ophthalmology, Pitié Salpêtrière University Hospital, Sorbonne Université, 75013 Paris, France; adam_mainguy@hotmail.fr (A.M.); bahram.bodaghi@aphp.fr (B.B.); 3 2nd Department of Ophthalmology, National and Kapodistrian University of Athens, 10679 Athens, Greece; eirchat@yahoo.gr; 4 Department of Ophthalmology and Visual Sciences, University of British Columbia, Vancouver, BC V6T 1Z4, Canada; kaivon9@gmail.com; 5 Department of Ophthalmology, Puerta de Hierro-Majadahonda University Hospital, Majadahonda, 28222 Madrid, Spain; jorge.ruizmedrano@gmail.com; 6 Department of Ophthalmology, Tel-Aviv Sourasky Medical Center Tel-Aviv, Israel Sackler Faculty of Medicine, Tel-Aviv University, Tel-Aviv 6997801, Israel; anatl@tlvmc.gov.il; 7 Center for Advanced Vision Science, Department of Ophthalmology, Department of Pathology, Department of Microbiology, Immunology, and Cancer Biology, University of Virginia School of Medicine, Charlottesville, VA 22908, USA; jambati@yahoo.com; 8 Department of Ophthalmology, University of Leiden, 2333 ZA Leiden, The Netherlands; 9 MIOS—Retina and Ocular Inflammation Center, 1005 Lausanne, Switzerland; mddesmet1@mac.com

**Keywords:** age-related macular degeneration, macular hemorrhage, macular hematoma, treat and extend, prognosis, retinal arterial macroaneurysm, vitrectomy

## Abstract

Objective: The study aimed to determine the outcomes and prognostic factors of vitrectomy, subretinal injection of tissue-plasminogen activator and gas tamponade in macular hemorrhage (MaH) due to age-related macular degeneration (AMD) or retinal arterial macroaneurysm (RAM). Methods: The study design utilized a multicentric retrospective case series design of consecutive patients undergoing surgery between 2014 and 2019. Results: A total of 65 eyes from 65 patients were included in the study. Surgery was performed after a mean period of 7.1 days. Displacement of MaH was achieved in 82% of the eyes. Mean best-corrected visual acuity (BCVA) improved from 20/500 to 20/125 at month(M)1 and M6 (*p* < 0.05). At M6, BCVA worsening was associated with an older age at diagnosis (*p* = 0.0002) and higher subretinal OCT elevation of MaH (*p* = 0.03). The use of treat and extend (TE) (OR = 16.7, *p* = 0.001) and small MaH fundus size (OR = 0.64 and 0.74 for horizontal and vertical fundus size, *p* < 0.05) were predictive of a higher likelihood of obtaining a countable BCVA at M1. Baseline BCVA was predictive of postoperative BCVA (*p* < 0.05). Retinal detachment and MaH recurrence occurred in 3% and 9.3% of cases at M6. Conclusion: MaH surgery stabilizes or improves BCVA in 85% of cases. Younger age at diagnosis, better baseline BCVA figures, smaller subretinal MaH height and use of TE regime were predictive of the best postoperative outcomes.

## 1. Introduction

Macular hemorrhage (MaH) is a serious and blinding complication of several pathologies, the most frequent being age-related macular degeneration (AMD). Other causes include any etiology of macular neovascularization (MNV), retinal arterial macroaneurysms (RAM), Terson syndrome, trauma, hematological conditions and idiopathic bleeding. When located in the subretinal space, the hematic collection can, within hours, cause irreversible damage to the photoreceptors due to iron-mediated toxicity, limitation of metabolic exchange (nutrients, oxygen, waste products) and clot contraction [1,2,3]. This toxicity might explain the poor prognosis experienced by some patients despite prompt management. Macular hematoma can also be located in the sub retinal pigment epithelium (RPE) space, decreasing visual acuity in case of large pigment epithelium detachments (PED) or RPE rips [4].

Several therapeutic approaches aimed at displacing the blood from the fovea have been described. Those include anti vascular endothelial growth factor (VEGF) injections, intravitreal injection of expansile gas, intravitreal injection of recombinant-tissue plasminogen activator (r-tPA) and gas (±anti-VEGFs), and surgery with variable outcomes depending on the technique [5,6,7]. The most widely used is complete pars plana vitrectomy (PPV) after induction of posterior vitreous detachment (PVD), subretinal injection of r-tPA, intravitreal filling with gas, and pre and post-operative injections of anti-VEGF agents. According to Boiché et al. [8], early surgical management using this technique results in an 81% displacement rate, and visual acuity improvement in 76.9% of treated eyes. 

While the risk factors of macular hemorrhage have been repeatedly suggested to include (at least in AMD) systemic hypertension, antiplatelet and anticoagulant intake and possibly the anti-VEGF therapy itself [3], the prognostic factors of visual improvement after surgical therapy have been described only in a few publications [4,8,9]. The role of pro-active regimes in AMD has not been scrutinized in this context. The purpose of this study was to determine the prognostic factors (clinical, ancillary and therapeutical) of favorable surgical outcome in patients with macular hemorrhage due to AMD or RAM. 

## 2. Methods

This was a multicenter retrospective observational clinical case series of consecutive patients recruited in three tertiary retina care units between December 2014 and December 2019, namely Lariboisière University Hospital, Paris, France, Puerta de Hierro-Majadahonda University Hospital, Madrid, Spain and the University of British Columbia, Vancouver, Canada. 

Consecutive adult patients with neovascular AMD or RAM complicated by macular hematoma (MaH) (size on funduscopy > 1.5 disc diameter) and managed surgically (see next paragraph) were included. To be included in the study, the MaH had to be located in the foveal area and involve the subretinal space (±sub-RPE) in AMD eyes; and the pre and/or subretinal space (±sub-RPE) in RAM eyes. The fovea was defined as the circular zone with a diameter of approximately one-disc centered on the macula. 

In addition to AMD, RAM patients were included because they have a theoretically healthy retina and thus form a comparative group allowing a better understanding of any causal parameter in AMD.

**Figure 1:**
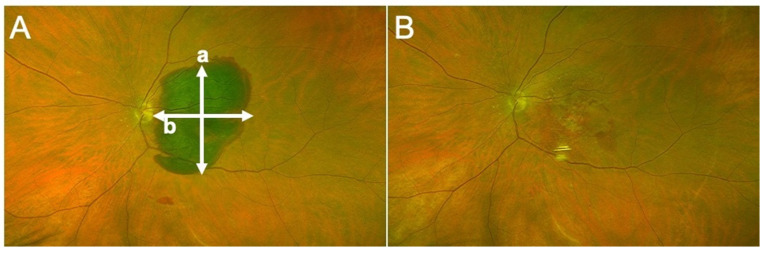
(**A**) Fundus photograph of a 70-year-old female with macular hematoma of her left eye, due to type 1 choroidal neovascularization consecutive to age related macular degeneration. (a) Vertical diameter, (b) horizontal diameter. (**B**) Surgery was performed 4 days after presentation, allowing complete displacement of blood from the foveal region.

**Figure 2:**
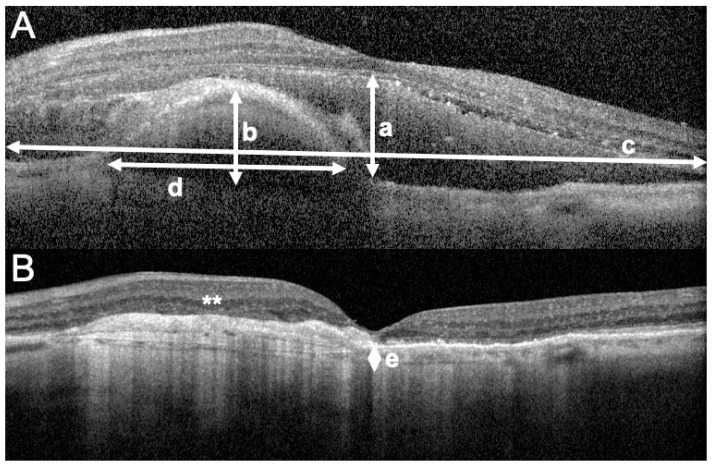
Optical coherence tomography (OCT) B scans passing by the foveal center, representing the left eye of an 81-year-old female presenting with macular hematoma consecutive to type-1 choroidal neovascularization due to age related macular degeneration. (**A**) OCT picture at the time macular hematoma occurrence. The height (a) and width (c) of the subretinal portion of the hemorrhage are represented with white arrows. When larger than the scale of the OCT B-scan, the maximum visible width is measured (c). The height and width of the sub-retinal pigment epithelium (RPE) portion of the hematoma are represented in (b) and (d). The size of the sub RPE portion of the hematoma is measured on the same B-scan as the subretinal portion of the hemorrhage. (**B**) OCT picture taken 6 months after surgery, showing complete displacement of the hematoma, the development of fibrotic lesions (white asterisks, **) at the level of the preoperative subRPE hemorrhage and atrophy of the photoreceptor layer. The post-operative B scan is obtained as a reference for the measurement of the subfoveal choroidal thickness (e). The pre-operative best corrected visual acuity was 0.05 decimals improving to 0.14 decimals at 6 months of follow-up.

All patients fulfilling the above-mentioned criteria during the study period were included. For all participants, demographic data, medical history, history of drug intake (more particularly, antiplatelet and/or anticoagulants) and previous anti-VEGF therapy, number of injections and regime (when applicable) were collected. Data of a standard ophthalmologic examination was retrospectively collected from before the occurrence of MaH, at baseline (at the time of presentation with MaH), and post-operatively (month 1, and 6). Collected data included best-corrected visual acuity (BCVA, decimals, converted to the logarithm of the minimum angle of resolution (logMAR) for statistical analyses), slit-lamp evaluation (lens status), measure of intraocular pressure (IOP), fundus color photograph (Optos California^®^, Dunfermline, UK) and spectral domain optical coherence tomography (SD-OCT) measurements (Spectralis, Heidelberg Instruments, Heidelberg, Germany; Cirrus, Zeiss, Jena, Germany) when available. With fundus photography, we measured the height and width of MaH (Figure 1). With SD-OCT, we measured the retrofoveal (from the central horizontal B-scan containing the most prominent foveal depression, in the central 1 mm-diameter) height and width of subretinal hemorrhage (SH), subRPE hemorrhage (SRH), and choroidal thickness (CT) by using the manual measure distance option of the manufacturer software (Figure 2). If the SH or SRH was higher or larger than viewable on the OCT B-scan, the maximal presentable height and/or width were measured.

For all patients, the surgery and potential complications were explained, and informed consent was obtained at the time of surgery. The protocol of the study and the procedures used were approved by the Ethics Committee of the French Society of Ophthalmology (Société Française d’Ophtalmologie, IRB 00008855, IRB number 1). This research adhered to the tenets of the Declaration of Helsinki. All patients underwent surgery under peribulbar or general anesthesia. Complete PPV (23 or 25G) after including (when needed) the induction of PVD, was performed in all participants. A gentle subretinal injection of rtPA (Actilyse^®^, Boehringer Ingelheim, Germany), diluted in BSS for a final concentration of 10 micrograms/mL, through a 41 G subretinal flexible canula (covering the entire size of MaH) was then performed covering at least the surface of the hematoma (D.O.R.C., Zuidland, Netherlands). Complete fluid–air exchange followed by gas injection (20%-SF6 or 17% C2F6) to a complete intravitreal fill was then performed. Phakic patients underwent standard small-incision cataract surgery when deemed necessary during the surgery or during the follow-up. Patients were instructed to keep a prone position for at least 4 h followed by a sitting vertical position for at least 2 days postoperatively. Postoperative anti-VEGF was administered monthly for at least 6 months (Ranibizumab 0.5 mg, Lucentis^®^, Novartis, Germany, or Aflibercept 2 mg, Eylea^®^, Bayer, Germany).

## 3. Statistical Analysis

Statistical analysis was performed using R software (R Core Team (2020). R: A language and environment for statistical computing. R Foundation for Statistical Computing, Vienna, Austria. URL https://www.R-project.org/.) and GraphPad Prism 6 (GraphPad Software, La Jolla, CA, USA). Quantitative variables were described using mean and standard deviation (SD) values, and categorical variables were described using absolute and relative frequencies. The vision was considered stable or improved after surgery; if BCVA was at least equal to the preoperative BCVA value. For prognostic factor determination, we used two surrogate outcomes. We presented the univariate analysis for obtaining a countable visual acuity (i.e., ≥ 20/400) at month 1. We also compared two groups (stable or improved BCVA versus worsened BCVA at month 6) using the appropriate t- and chi-squared tests. For patients with no available BCVA at month 6, the latest available data was used according to the last observation carried forward (LOCF) model. Correlations between continuous quantitative variables were performed by calculating the Spearman correlation coefficients (r). The level of significance was set at *p* < 0.05.

## 4. Results

Patient characteristics are described in Table 1. Sixty-five eyes of 65 patients were included. There were 57 eyes with MaH due to AMD (group 1) and eight eyes with MaH due to RAM (group 2). The mean age of the cohort was 78.7 ± 9.6 years (range: 55–95). The percentage of females was 53.9%. Overall, patients in both groups were comparable in terms of demographics (age, gender) and medical history (systemic hypertension, diabetes) (all *p* > 0.05). Patients with RAM were more frequently on antiplatelet therapy (*p* = 0.04). Among AMD eyes, 81.4% were related to occult MNV. AMD eyes were treated with anti-VEGF (mean number of injections prior to MaH = 8.8 ± 9.5; median = 4) following mostly a *Pro Re Nata* (PRN) regime (Supplementary Table S1 and Table 3).

Before MaH, the mean BCVA was higher in the RAM group (20/25 Snellen; 0.81 ± 0.29 decimals) versus the AMD group (20/50 Snellen; 0.45 ± 0.27 decimals, *p* = 0.03). At first presentation with MaH, the mean BCVA decreased dramatically in both groups, to 20/400 (0.05 ± 0.08 decimals) in group 1 and hand motion (0.01 ± 0.02 decimals) in group 2.

On fundus photographs, the average vertical and horizontal diameters of the hematoma were comparable between group 1 and 2 (*p* > 0.05). On OCT, all AMD eyes (100%) had subfoveal subretinal hematoma (SH) versus 75% in the RAM eyes (*p* = 0.01). None of the AMD eyes had preretinal hematoma versus 25% (N = 2) in the RAM eyes. The two RAM eyes that had preretinal hematoma underwent rapid surgery because they were also suspected to have subretinal hematoma on pre-operative OCT. Absence of subretinal hematoma was noted only per-operatively. Subfoveal subRPE hematoma (SRH) was present in 91.2% of AMD eyes versus 12.5% of RAM eyes (*p* < 0.0001). No RPE rips were noted during the available follow-up.

On OCT, the mean size of the subfoveal SH was slightly higher in AMD (vertical size 490.9 ± 237.4 microns, horizontal size 5441.3 ± 1323.1 microns) versus RAM eyes (vertical size 385.1 ± 429.2 microns, horizontal size 5085.2 ± 3012.3 microns), but this was not statistically significant (all *p* > 0.05). Retrofoveal choroidal thickness (CT) was comparable between both groups (same age range in both groups).

All patients were managed surgically after a mean period of 7.1 ± 6.7 days (range 0–30 days), with no difference being noted between group 1 and 2. At month 1, surgical intervention resulted in maintenance or improvement of vision in 84.1% of cases (85.4% in the AMD group, 75% in the RAM group). At month 6, vision was maintained or improved in 84.6% of cases (82.3% in the AMD group, 100% in the RAM group). We found an overall anatomical success rate of 82% (complete displacement of MaH on fundus photographs at M6).

Surgical complications occurred in 7.6% of cases at 1 month: intravitreal hemorrhage (6.1% of cases; N = 4:1 in the AMD group and 3 in the RAM group), rhegmatogenous retinal detachment (1.5%; N = 1 in the AMD group and 0 in the RAM group); and 12.3% of cases at 6 months: rhegmatogenous retinal detachment (3% of cases; N = 2:1 in the AMD group and 1 in the RAM group); and recurrence of hematoma (9.3% of cases; N = 6:5 in the AMD group and 1 in the RAM group). No case of a macular hole was reported.

We looked at the predictive factors for obtaining a “countable” BCVA at M1 (i.e., ≥20/400 Snellen, ≤1.3 LogMar, Table 2). At month 1, 54% of patients had a countable BCVA versus 27% at baseline. Using univariate analysis, younger age (odds ratio (OR) = 0.9, *p* = 0.05) at MaH diagnosis, high baseline BCVA (OR = 4.34, *p* = 0.011), presence of a countable BCVA at baseline (OR = 4.37, *p* = 0.02), smaller horizontal (OR = 0.64, *p* = 0.004) and vertical (OR = 0.74, *p* = 0.006) size of MaH on fundus photography, and previous use of TE regime in AMD eyes (OR = 16.7, *p* = 0.001) were correlated with a higher likelihood of obtaining a countable BCVA at M1. The baseline BCVA was higher in eyes that reached a countable BCVA at M1 versus those that did not (1.55 logMAR versus 1.90 LogMAR, *p* = 0.008). Baseline BCVA was predictive of postoperative BCVA (see Supplementary Materials: Figures S1 and S2 at M1 (r = 0.413, *p* < 0.0001) and M6 (r = 0.441, *p* = 0.006). On the other hand, the lens status has no effect on this BCVA outcome (*p* = 0.98).

Regarding the predictive factors of surgical outcomes at 6 months, eyes with BCVA stability or improvement were compared to those with BCVA worsening (Table 3, Supplementary Table S2). Of note, 83.9% of eyes were pseudophakic at M6 (82.4% in the AMD group and 87.5% in the RAM group, Table 1). Besides, 100% of eyes with worsened BCVA at M6 were pseudophakic, limiting the participation of lens status on BCVA outcomes. BCVA worsening at M6 was significantly associated with an older age at MaH occurrence (*p* = 0.0002), and higher OCT elevation of the subretinal portion of MaH (*p* = 0.03). Among AMD patients and although not statistically significant, patients whose visual acuity decreased at month 6 had more frequently been treated with a PRN regime (80%) versus those whose BCVA was stable or improved (50%). Although not statistically significant, patients whose visual acuity decreased at month 6 had surgery later than eyes with BCVA stability or improvement (average of 10 days versus 6.8 days). Among eyes with subretinal MaH (N = 63), the proportion of those with BCVA stability or improvement at M6 did not differ depending on the etiology (AMD or RAM) (*p* = 0.98).

## 5. Discussion

The risk factors for MaH occurrence have been validated in multiple studies and include systemic hypertension and anticoagulant intake [3]. On the other hand, publications questioning the prognostic factors of visual acuity improvement after surgical treatment remain scarce in the context of MaH. More specifically, the implication of anti-VEGF treatment regime in the final prognosis has never been investigated.

Previous papers suggest that a large MaH area, increased retinal elevation and diagnosis of AMD are factors of poor visual outcome. However, not all studies have investigated the results of surgical management, and among the surgical cohorts, many have described the use of invasive techniques (such as clot aspiration) associated with more frequent surgical complications [3,10,11]. In accordance with a recent review [3], we use 23 or 25 G PPV, subretinal injection of tPA, gas tamponade (non-expansile), per-operative and subsequent monthly postoperative anti-VEGFs (for at least 6 months) to treat large MaHs with vision impairment, because this has been shown to be more effective in reducing the size of the scar than pneumatic displacement alone, with or without intravitreal rtPA [6,11,12,13,14,15]. We therefore aimed to investigate the clinical, ancillary and treatment-related factors of visual acuity improvement or worsening using the aforementioned surgical technique in MaH complicating AMD or RAM. Although the sample was limited, RAM eyes were used as internal controls for AMD eyes.

The demographic characteristics of our patients were similar to those reported in other publications using the same surgical techniques [4,8,9]. Our patients had a mean age of 78 years, and we had a slightly higher proportion of females. The majority of MaH eyes were secondary to AMD (in Plemel et al., 84.6% of included eyes were due to AMD, and 5.1% were due to RAM) [9]. Patients were managed surgically after a mean period of 7.1 days, which was in the same range as other similar publications (mean of 6.4 days in Plemel et al. [9] and 4.9 days in Treumer et al. [4,16]). The percentage of patients on anticoagulants or antiplatelet therapy was also comparable with other studies [4,8]. We found an anatomical success rate (complete displacement of MaH) of 82% (81% in Boiché et al.) [8] and visual acuity stabilization or improvement in 84.6% of cases at month 6, which was comparable with other publications [17,18]. Surgical complications occurred in 7.6% of cases at 1 month (intravitreal hemorrhage (IVH), rhegmatogenous retinal detachment (RRD)); and in 12.3% of cases at 6 months (RRD, recurrence of MaH). The rate of IVH and RRD was comparable to what was previously described [16]. However, our recurrence rate was lower (9.3% of cases in the present paper versus 20% in Treumer et al. and 29% in Gonzalez-Lopez et al.) [19]. This can possibly be explained by the fact that anti-VEGFs were used following a monthly regime in the post-operative period in all included eyes [8]. We did not report on any case of macular hole. RPE rips represent another important complication of macular hematomas and their surgical management. It has been shown that RPE rips tend to occur more frequently in eyes with PED heights > 500 microns [4,20,21,22]. We recorded no RPE tears during the follow-up time of this study.

Regarding the prognostic factors of post-operative visual acuity outcomes, we were able to confirm previous findings: the characteristics of hemorrhagic PED on OCT, and time between presentation and surgery were not correlated with BCVA status (worsening or improvement) at 6 months [12,23,24]. Regarding the time to surgery, the absence of correlation was probably due to the fact that all patients were operated within 30 days of their first symptoms, decreasing the power of statistical analyses. Recurrence of macular hematoma during the follow-up was correlated with a poor visual prognosis (mean BCVA at 6 months was 0.025 decimals in eyes with recurrent MaH versus 0.20 decimals in those with non-recurrent MaH, *p* = 0.001) but this was not correlated with the use of anticoagulants or antiplatelet therapy, as reported by others [9].

We also report on several new findings in this study; we first showed that older patients were at higher risk of visual acuity loss after surgery. Interestingly [9], among eyes with subretinal hematoma, the etiology of MaH was not predictive of the visual outcome. In fact, the proportion of AMD and RAM eyes was similar between the group with BCVA stability/improvement (87.7% of AMD eyes) and the group with BCVA worsening at 6 months (87.5% of AMD eyes). An older age might therefore be an independent relevant factor of poor prognosis, regardless of MaH etiology. We speculate that retinal cells of elderly patients might be more susceptible to iron toxicity than those of younger individuals.

Contrary to several previous studies addressing the same topic [8,9], we found that the height of the subfoveal subretinal portion of MaH was correlated with the final BCVA outcome. In fact, eyes with the highest subretinal MaH elevation on OCT were at higher risk of BCVA worsening at 6 months (*p* = 0.03). An arbitrary cut-off of > 520 microns was found to yield a 77.7% sensitivity and a 71.4% specificity rate for visual acuity worsening at month 6 (true positives: 7 of 9 eyes, true negatives: 15 of 21). This particularly interesting finding could be explained by the fact that higher hematomas might slow and limit the diffusion of nutrients and oxygen molecules to photoreceptor cells, and that of waste products, which would otherwise pass between the retina and the retinal RPE, thereby precipitating cell death and atrophy. We however could not find any difference between the postoperative rate of macular atrophy in eyes with subretinal MaH height > or <520 microns. Although arbitrary, and not based on an ROC curve (small sample), this 520-micron cut-off might help surgeons provide prognostic information to their patients.

Contrary to Plemel et al. [9], we did not find any correlation between the distance from the fovea to the superior, inferior, nasal or temporal edge of MaH, and visual acuity at 6 months (data not shown). In fact, the authors suggested that macular hematomas with larger portions in the superior part of the fundus might be more difficult to displace inferiorly. We tended to inject rtPA volumes that cover the entire MaH surface, which could help drain the blood and explain our findings. On the other hand, we found that smaller vertical and horizontal sizes of MaH (on fundus photographs, Figure 1A) were correlated with a higher likelihood of obtaining a countable post-operative BCVA. We found in contradiction with others, that baseline BCVA was linearly correlated with BCVA value at 1 and 6 months, independent of lens status since the majority of eyes were pseudophakic by M6 (Table 2 and Table 3).

Although not statistically significant, we reported a higher proportion of eyes treated with a PRN regime in the group of AMD eyes with BCVA worsening versus the group with BCVA stability or improvement at 6 months. Univariate analyses confirmed that PRN (versus TE) was correlated with poor visual outcomes: the odds ratio for obtaining a countable visual acuity at month 1 in the group of AMD eyes previously treated with a TE regimen (versus PRN) was 16.7, *p* = 0.001. This was never reported elsewhere and constitutes additional data supporting the use of proactive strategies in the treatment of AMD.

There are limitations to this study that should be mentioned. They include its retrospective nature, the limited sample size especially in the RAM group, the potential small variations in surgical techniques between surgeons and the difficulty in measuring the dimensions of MaH in some of the included eyes. While we cannot retrospectively check for small variations in surgical techniques, we believe that they are slight enough not to prevail in the final visual outcome. On the other hand, including patients from only one surgeon would induce a more important bias, limiting the external validity of the study. While ideal measurements would have included a volumetric assessment of the subretinal and subRPE portions of MaH, our two-dimensional findings should still help surgeons inform their patients about their expected prognosis. Patient demographic characteristics were comparable between the three tertiary centers with no center-related effect being detected. While previous papers mentioned it, we did not report on the predictive value of identifying a visible or obscured ellipsoid layer on preoperative OCT [25,26]. Data regarding the proportion of eyes with remaining sub or intraretinal fluid and subretinal fibrosis is also lacking.

This work was exploratory and used only univariate analyses. Yet, this cohort is one of the largest ever published on this topic. We believe that future collaborative studies with larger sample sizes would be helpful to better understand the implications of our findings.

## 6. Conclusions

In conclusion, we showed that PPV with subretinal rtPA injection, intravitreal gas filling and anti-VEGF injection, was a valid and safe therapeutic option in MaH and visual acuity loss. Older age at diagnosis and higher subretinal MaH elevation are correlated with visual acuity worsening TE regime in the case of AMD eyes correlate with a higher likelihood of obtaining countable postoperative visual acuities.

## Figures and Tables

**Table 1:** Patient demographic and clinical characteristics.

	AMD (*n* = 57 ^a^) (Mean ± SD; Range) or N (%)	RAM (*n* = 8 ^a^) (mean ± SD; Range) or N (%)	*p*-Value
Age (years)	79.5 ± 9.1 (58–95)	73 ± 11.3 (55–85)	0.15
Gender (females)	31 (54.4%)	4 (50%)	1
Antiplatelet intake	9 (18%)	4 (66.7%)	0.04
Systemic hypertension	35 (67.3%)	5 (83.3%)	0.42
Diabetes	8 (14.8%)	1 (20%)	0.90
Antiglaucoma drops	6 (11.5%)	1 (25%)	0.59
Previous PDT or laser therapy	4 (7.8%)	-	-
CNV Type:			-
- Occult	44 (81.4%)		
- Visible	1 (1.9%)		
- Polypoidal	9 (16.6%)		
Mean BCVA (decimals):			
- Before MaH	0.45 ± 0.27 (0.01–1)	0.81 ± 0.29 (0.3–1)	0.03
- Baseline (at the time of MaH)	0.05 ± 0.08 (0.005–0.05)	0.01 ± 0.02 (0.005–0.02)	0.02
- M1 postoperative	0.13 ± 0.15 (0–0.6) (*p* < 0.001 vs. Baseline)	0.31 ± 0.41 (0–1) (*p* < 0.05 vs. Baseline)	0.25
- M6 postoperative	0.13 ± 0.19 (0.005–0.67) (*p* < 0.001 vs. Baseline)	0.40 ± 0.49 (0.02–1) (*p* < 0.05 vs. Baseline)	0.29
Lens status (pseudophakic)			
- Baseline (at the time of MaH)	26 (45.6%)	5 (62.5%)	0.37
- M1 postoperative	34 (59.6%)	5 (62.5%)	0.87
- M6 postoperative	47 (82.4%)	7 (87.5%)	
Fundus photography			
- Vertical size of MaH (mm)	7.7 ± 3.6 (2.4–18.3)	7.6 ± 1.9 (5.6–10.3)	0.78
- Horizontal size of MaH (mm)	7.4 ± 3.9 (2.5–24.3)	7.1 ± 1.8 (4.6–9.5)	0.72
OCT			
Retrofoveal subretinal hematoma:	57 (100%)	6 (75%)	0.01
- Vertical size (microns)	490.9 ± 237.4 (116–1149)	385.1 ± 429.2 (0–1088)	0.54
- Horizontal size (microns)	5441.3 ± 1323.1 (2629–8596)	5085.2 ± 3012.3 (0–7636)	0.80
Retrofoveal sub-RPE hematoma	52 (91.2%)	1 (12.5%)	<0.0001
- Vertical size (microns)	467.4 ± 269.4 (0–1234)	681	-
- Horizontal size (microns)	3265.7 ± 1540.5 (0–6852)	3621	-
Retrofoveal CT (microns)	224.7 ± 169.8 (81–1078)	274.6 ± 127.8 (119–465)	0.42
Time before surgery (days)	7.1 ± 6.3 (0–28)	6.75 ± 9.6 (1–30)	0.91

AMD: age-related macular degeneration, RAM: retinal arterial macroaneurysm, N = number, %: percentage, PDT: photodynamic laser therapy, CNV: choroidal neovascularization, BCVA: best-corrected visual acuity, MaH: macular hematoma, M: month, OCT: optical coherence tomography, RPE: retinal pigment epithelium, CT: choroidal thickness. mm: millimiter. Retrofoveal measures were performed on a horizontal B scan passing by the central fovea, in the central 1 mm. ^a^ *n* = 57 for AMD and 8 for RAM unless indicated otherwise through percentages.

**Table 2:** Predictive factors of obtaining a countable visual acuity at one month of surgery.

Variable	Univariate Odds Ratio (CI, *p*-Value)
Age	0.94 (0.89–1.00, *p* = 0.05)
Sex (Female)	1.18 (0.44–3.22, *p* = 0.74)
Countable BCVA at baseline	4.37 (1.33–17.36, *p* = 0.021)
Baseline BCVA (LogMAR)	0.23 (0.07–0.67, *p* = 0.011)
Lens status at month 1	0.99 (0.35–2.75, *p* = 0.980)
Injection regime (Treat and extend) in AMD eyes	16.71 (3.51–126.60, *p* = 0.001)
Time to surgery	0.94 (0.86–1.02, *p* = 0.17)
Horizontal size of hematoma on fundus photograph	0.64 (0.45–0.83, *p* = 0.004)
Vertical size of hematoma on fundus photograph	0.74 (0.58–0.90, *p* = 0.006)

CI: 95% confidence interval.

**Table 3:** Predictive factors of best corrected visual acuity outcomes at 6 months.

BCVA at Month 6	Stable or Improved (N = 57) ^a^	Worsened (N = 8) ^a^	*p*-Value
Mean age (years)	77.5	85	0.0002
Mean baseline BCVA (decimals)	0.03	0.10	0.28
Fundus photography			
- Mean MaH vertical size (mm)	7.7	7.1	0.7
- Mean MaH horizontal size (mm)	7.6	6.8	0.5
OCT			
Subretinal hematoma			
- Mean vertical size (microns)	415.8	634.6	0.03
- Mean horizontal size (microns)	5082.4	6170.3	0.1
Sub-RPE hematoma			
- Mean vertical size (microns)	395.9	328.8	0.4
- Mean horizontal size (microns)	2790.4	3121.8	0.7
Mean subfoveal choroidal thickness (microns)	239.5	196.5	0.6
Mean number of days between MaH and surgery	6.8	10	0.4
Mean last known injection interval (weeks)	21.4	21.5	0.9
Anti-VEGF regime in AMD eyes (% of PRN) before hematoma	24 (50%)	4 (80%)	0.3

AMD: age-related macular degeneration, BCVA: best-corrected visual acuity, VEGF: vascular endothelial growth factor, N: number, MaH: macular hematoma, OCT: optical coherence tomography, RPE: retinal pigment epithelium, PRN: *pro re nata*, mm: Millimeter, %: percentage. ^a^ For eyes with unavailable BCVA data at M6, figures were extrapolated from the M1 or M3 endpoints (see methods section).

## Data Availability

The data presented in this study are available on request from the corresponding author.

## Supplementary Materials

The following are available online at https://www.mdpi.com/article/10.3390/jcm10245787/s1, Figure S1: Graphical representation showing the correlation between pre-operative best corrected visual acuity (BCVA) (x-axis) and post-operative BCVA at 1 month of surgery (y-axis) (Pearson correlation analysis, r = 0.413, *p* < 0.0001). Figure S2: Graphical representation showing the correlation between pre-operative best corrected visual acuity (BCVA) (x-axis) and post-operative BCVA at 6 months of surgery (y-axis). (Pearson correlation analysis, r = 0.441, *p* = 0.006). Table S1: History of anti-VEGF injections in AMD patients. Table S2: Predictive factors of best corrected visual acuity outcomes at 6 months in the AMD group.
