# Supplementary material for: Macular Hemorrhage Due to Age-Related Macular Degeneration or Retinal Arterial Macroaneurysm: Predictive Factors of Surgical Outcome
Source: J Clin Med. 2021 Dec 10;10(24):5787. doi: 10.3390/jcm10245787 (PMC8703651; doi:10.3390/jcm10245787)
Supplement: Supplementary file 1 [file jcm-10-05787-s001.zip › jcm-1472049-Supplymentary.pdf]

Supplementary Table S1: History of anti-VEGF Injections in AMD Patients (N = 57)	
	**AMD patients**<br>**Mean ± SD (Range) Or N(%)**
Number of anti-VEGF injections received prior to MaH	8.8 ± 9.5 (0–27)
Last known injection interval before MaH (weeks)	21.4 ± 36.6 (0–170)

AMD: Age-related macular degeneration, MaH: Macular hematoma, N:Number, %: Percentage, VEGF: Vascular endothelial growth factor.

Supplementary Table 2. Predictive factors of best corrected visual acuity outcomes at 6 months in the AMD group

BCVA at Month 6	Stable or improved (N = 51)[a]	Worsened (N = 6)[a]	*p*-value
Mean age (years)	78.4	84.3	0.06
Mean baseline BCVA (decimals)	0.03	0.13	0.20
Fundus photography			
- Mean MaH vertical size (mm)	7.9	6.2	0.31
- Mean MaH horizontal size (mm)	7.7	6.1	0.03
OCT			
Subretinal hematoma			
- Mean vertical size (microns)	435.6	593.6	0.04
- Mean horizontal size (microns)	5336.7	6138.3	0.26
Sub-RPE hematoma			
- Mean vertical size (microns)	468.2	544.5	0.55
- Mean horizontal size (microns)	3275.5	3348.0	0.98
Mean subfoveal choroidal thickness (microns)	197.5	248.7	0.48
Mean number of days between MaH and surgery	7.6	5.1	0.20
Mean last known injection interval (weeks)	23.6	8.8	0.20

AMD: Age-related macular degeneration, BCVA: Best-corrected visual acuity, VEGF: Vascular endothelial growth factor, N: Number, MaH: Macular hematoma, OCT: Optical coherence tomography, RPE: Retinal pigment epithelium, mm: Millimeter, %: Percentage.
[a]For eyes with unavailable BCVA data at M6, figures were extrapolated from the M1 or M3 endpoints (see methods section).

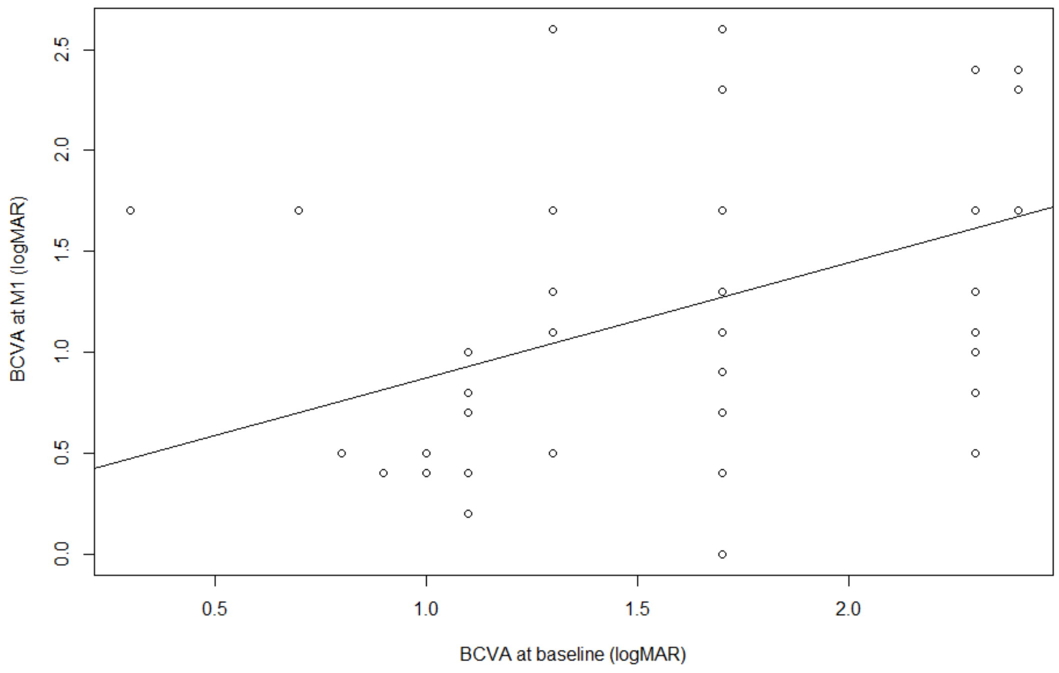

Figure S1. Graphical representation showing the correlation between pre-operative best corrected visual acuity (BCVA) (x-axis) and post-operative BCVA at 1 month of surgery (y-axis) (Pearson correlation analysis, r = 0.413, $p < 0.0001$).

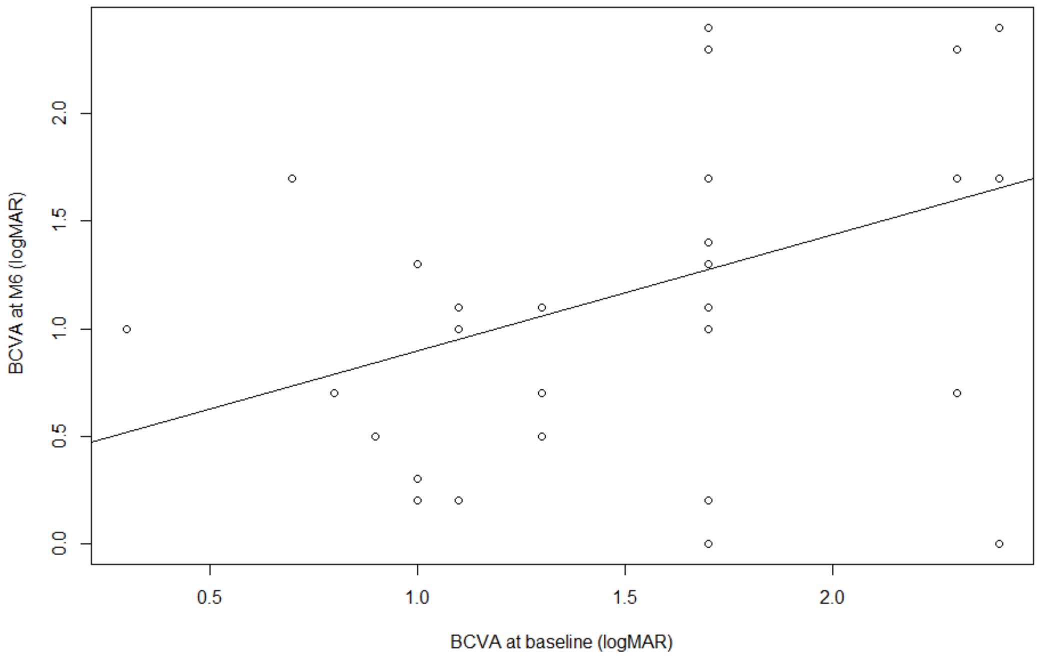

Figure S2. Graphical representation showing the correlation between pre-operative best corrected visual acuity (BCVA) (x-axis) and post-operative BCVA at 6 months of surgery (y-axis). (Pearson correlation analysis, r = 0.441, $p = 0.006$).
